# Thromboembolic Disease and COVID-19: Experience of a University and Emergency Hospital During the Pandemic

**DOI:** 10.7759/cureus.68767

**Published:** 2024-09-06

**Authors:** Miruna F Stefan, Lucia S Magda, Roxana C Rimbas, Dragos Vinereanu

**Affiliations:** 1 Cardiology and Cardiovascular Surgery, University and Emergency Hospital Bucharest, Bucharest, ROU; 2 Cardiology and Cardiovascular Surgery, Carol Davila University of Medicine and Pharmacy, Bucharest, ROU

**Keywords:** covid-19, emergency hospital, pandemic, pulmonary embolism, sars-cov-2, venous thromboembolic disease

## Abstract

Background: COVID-19 leads to vasculopathy, which is linked to both a prothrombotic state and an impaired immune response. A notable increase in pulmonary embolism (PE) and deep venous thrombosis (DVT) has been documented.

Methods: We conducted a retrospective analysis of all patients who were admitted with venous thromboembolic disease (VTD) in the largest university and emergency hospital in Romania, between May 1, 2020, and April 30, 2021. Patients were categorized into two groups based on the presence (Group 1) or absence (Group 2) of COVID-19 virus infection at the time of admission. The aim of this study was to assess the characteristics of VTD in COVID-19 patients and to compare the clinical and paraclinical parameters of the Group 1 and Group 2 patients admitted for VTD in an emergency hospital during the first two waves of the pandemic (12 months). We compared clinical, biological, and imaging parameters and applied binary logistic analysis for the predictive models.

Results: A total of 198 patients were diagnosed with VTD (at admission or during the hospitalization); out of 33,373 patients hospitalized, 43 (21.7%) were diagnosed with COVID-19 (12.2% with mild COVID-19, 61.0% moderate, and 26.8% severe). Group 1 showed higher heart rates and leukocytes, more severe pulmonary changes (p<0.05), higher N-terminal-pro-B-type natriuretic peptide (NTproBNP), and high sensitivity troponin I (hs-cTnI) (p>0.05). Not reaching statistical significance, the mortality tended to be higher in Group 1. These patients were admitted to the intensive care units for longer (3.5 vs. 1.5 days, p > 0.05). The minimum value of thrombocytes during hospitalization was inversely correlated with the risk of death. Interestingly, the Pulmonary Embolism Severity Index (PESI) score was not predictive for in-hospital death in Group 1, but only in Group 2 (area under the curve (AUC) = 0.821, CI 0.689-0.952).

Conclusion: Individuals with severe manifestations of COVID-19 remain vulnerable to developing VTD and are prone to adverse outcomes. The efficacy of PESI as a predictive tool for in-hospital death is non-significant. Further refinement of specific predictive scores tailored to VTD associated with COVID-19 is needed.

## Introduction

Since its first outbreak in Wuhan in December 2019, the infection with the novel coronavirus, known as COVID-19, has quickly spread around the globe, with more than 775 million individuals affected and nearly seven million deaths [1].

The COVID-19 infection shows a large spectrum of clinical manifestations, from asymptomatic or mild to multiple organ dysfunction, cytokine storms, thrombotic complications, and finally death. Among the most prevalent manifestations are fever, dyspnea, dry cough, and myalgia, followed by viral pneumonia and respiratory failure [2].

The virus uses the angiotensin-converting enzyme-2 (ACE2) protein receptors, which are mostly found in the lungs, heart, kidney, gastrointestinal tract, and blood vessels, to enter by endocytosis into the host cells [3]. Due to the high expression of the ACE2 receptors in type II alveolar cells, the virus causes diffuse alveolar damage and hyperinflammation, which subsequently leads to pneumonia and acute respiratory distress syndrome (ARDS). Moreover, a large spectrum of extrapulmonary manifestations has been reported, including cardiovascular, hematologic, and thrombotic complications [3].

The vasculopathy observed in association with COVID-19 isn’t just an innate prothrombotic effect of the virus but also a consequence of the immune response of the host, through the release of pro-inflammatory mediators such as interleukin-1 (IL-1), IL-6, and tumor necrosis factor-a (TNF-a) that interact with the platelets and stimulate the expression of tissue factor (high-affinity receptor and cofactor for factor (F)VII/VIIa), eventually initiating the extrinsic coagulation cascade. This results in a suppression of the fibrinolytic system and produces an upregulation of plasminogen activator inhibitor-1 [2].

Consequently, a high incidence of pulmonary embolism (PE) and deep venous thrombosis (DVT) has been reported in COVID-19. Moreover, microthrombi located in many organs (lungs, heart, kidneys) have been described in histological postmortem specimens. In severe illness, micro-thrombosis might lead to a generalized coagulopathy [4].

The incidence and prevalence of venous thromboembolic disease (VTD) in COVID-19 vary greatly in the literature. One large meta-analysis, including 48 studies with 13,824 patients, reports overall prevalence rates of PE and DVT around 7.8% and 11.2%, respectively, with even higher values for critically ill patients (23.2%), which is higher than the one reported in the general population, where the condition has an estimated prevalence of approximately 1 in 1,000 individuals [4-5].

Another meta-analysis of 42 studies involving 8,271 COVID-19 patients has shown that the overall VTD incidence was 21%, with a DVT rate of 20% and a PE rate of 13%. Among critically ill patients, the VTD rate was even higher (31%), the DVT rate was 28%, and the PE rate was 19%. This may be due both to the severity of the disease and the systemic inflammation associated with it, as well as the immobilization [6]. It was shown that COVID-19-positive patients that were admitted to the intensive care unit presented with persisting hypercoagulability and hypofibrinolysis, despite anticoagulant treatment [7].

Therefore, the aim of this study was to assess the characteristics of VTD in COVID-19 patients and to compare the clinical and paraclinical parameters of the COVID-19-positive and COVID-19-negative patients admitted for VTD in an emergency hospital during the first two waves of the pandemic (12 months). Moreover, we tried to identify predictive parameters that could estimate the outcome of COVID-19 patients with associated VTD.

Of mention, at the beginning of the pandemic, the healthcare system in our country was organized to manage the influx of COVID-19 patients. All infectious disease hospitals were designated to admit SARS-CoV-2-positive patients. Other hospitals, including those specializing in chronic care, were designated as "support" facilities for these primary COVID-19 hospitals. As the largest emergency hospital in Romania, our facility underwent a significant reorganization to handle the crisis. This reorganization included changes from the emergency department to all units within the hospital. Each unit was divided into three sections with separate circuits to limit the spread of the virus. The units were, 1. A "buffer" zone where patients were isolated in individual chambers while receiving medical care and awaiting real-time reverse transcriptase-polymerase chain reaction (RT-PCR) test results collected in the emergency department; 2. A dedicated area for patients who tested positive for SARS-CoV-2; 3. Another area for patients who tested negative. Our hospital admitted all patients considered medical emergencies, regardless of their SARS-CoV-2 status, and provided the necessary diagnosis and management for various cardiac emergencies.

## Materials and methods

Study design

The aim of this study was to assess the characteristics of VTD in COVID-19 patients and to compare the clinical and paraclinical parameters of COVID-19-positive and COVID-19-negative patients admitted for VTD in an emergency hospital during the first two waves of the pandemic (12 months). We retrospectively identified all patients hospitalized in the University and Emergency Hospital, Bucharest, Romania, the largest emergency hospital in the country, between May 1, 2020, and April 30, 2021. We selected the patients who were hospitalized for VTD either in the cardiology department or in all the other departments (internal medicine, intensive care unit, surgery, orthopedics, obstetrics & gynecology, etc.). A PE was diagnosed using computed tomography pulmonary angiogram (CTPA) and was defined as at least one filling defect present at the level of the pulmonary arterial tree. A DVT was diagnosed using lower extremity venous ultrasonography. Data were collected from the hospital registries by doctors. Information was extracted from both electronic health records and observational paper records available at the hospital. The process was standardized to maintain consistency. The inclusion criteria were: age more than 18 years old; symptoms and signs suggestive of VTD, confirmed by venous Doppler ultrasonography or/and by CTPA; RT-PCR test that either confirmed or disproved the SARS-CoV-2 infection. The exclusion criteria were: patients with incomplete data and recent recovery from SARS-CoV-2 infection (less than one month). We divided the patients into two subgroups: patients positive for the SARS-CoV-2 infection (Group 1) and patients negative for the SARS-CoV-2 infection (Group 2) at the time of admission. The COVID-positive or negative status was assessed using real-time quantitative RT-PCR assays on nose and throat swabs, which were routinely performed for all patients admitted to our hospital. 

Initial assessment

We have considered demographic, clinical, and biological parameters, the cardiovascular diagnosis, the clinical status at presentation, the presence of symptoms suggestive of SARS-CoV2 infection, the number of days between the onset of symptoms and hospitalization, the severity of the COVID-19 disease according to the epidemiological definitions at the time, the Pulmonary Embolism Severity Index (PESI), medical history of the patients and predisposing factors for venous thromboembolism, the existence of another concomitant infection, the medication that was administered (anticoagulants and medication commonly used to treat COVID-19), pulmonary imaging, vascular and cardiac ultrasound and electrocardiographic parameters. 

Demographic and Clinical Data

These included age, gender, weight, height, and body mass index; heart rate (at admission and mean during hospitalization); systolic and diastolic blood pressures (at admission and mean during hospitalization); atrial and ventricular arrhythmias; symptoms (cough, myalgia, dysphagia, ageusia, anosmia, dyspnea, digestive symptoms); pulmonary auscultation; fever; highest temperature during hospitalization; arterial oxygen saturation. We defined low-grade fever as a temperature between 37.3°C to 38.0°C and high-grade fever as a temperature above 39.1°C, in accordance with the data in the literature. Doctors measured all vital signs upon patient admission, during each morning's medical rounds, and whenever there was a change in the patient's condition.

Medical History

We recorded the medical history of the patients and most important risk factors for venous thromboembolism, including smoking status (current smokers being defined as individuals who have smoked at least 100 cigarettes in their lifetime and report that they smoke either daily or occasionally), stroke history, heart failure, hypertension, dyslipidemia, coronary disease, valvular disease, chronic obstructive pulmonary disease (COPD), peripheral arterial disease, chronic kidney disease, neoplasia, recent surgery, thrombophilia, chronic venous insufficiency, recent fracture, bed immobilization, dementia, pregnancy, childbed, and history of thromboembolism. 

Conventional Biological Data

This included admission hemoglobin level, number of leukocytes and the leukocyte formula, highest leukocyte number during hospitalization, thrombocyte number at admission, highest value of erythrocyte sedimentation rate (ESR), C-reactive protein (CRP) and fibrinogen, ferritin, highest high-sensitivity troponin I level, admission NTproBNP, aspartate aminotransferase (AST), alanine aminotransferase (ALT), admission blood urea nitrogen (BUN), admission creatinine, admission eGFR (estimated glomerular filtration rate), glycated hemoglobin (HbA1c), creatine kinase (CK), creatine kinase myocardial band (CK-MB), and lactate dehydrogenase (LDH). A standard 12-lead electrocardiogram was performed on admission and repeated during hospitalization for all patients. The occurrence of atrial and ventricular arrhythmias was assessed.

Echocardiographic Data

This included left ventricular ejection fraction (LVEF), paradoxical interventricular septal motion, right atrioventricular pressure gradient (derived from the spectral Doppler of the tricuspid regurgitation jet), and tricuspid annular plane systolic excursion (TAPSE).

Pulmonary Imaging

We recorded accentuation of the interstitial pattern, pulmonary consolidation or alveolitis on the chest X-ray, ground-glass image, its location, aspects suggestive of acute respiratory distress syndrome (ARDS), and/or the presence of subpleural condensation on computed tomography. After intravenous contrast administration, the pulmonary arterial vasculature was checked for filling defects. All images were analyzed by the radiology department in our hospital.

Venous Doppler Ultrasound

The presence of venous thrombi or lack of normal venous compressibility was noted. All investigations were performed by physicians with expertise in this type of examination. 

Follow-up

We collected data regarding the treatment received by the patients, both during hospitalization and at discharge. We used a previous article by Canale et al. to help us stratify the positive patients for the SARS-CoV-2 infection into mild form, moderate form, and severe form. Mild illness was defined by general viral symptoms and may include gastrointestinal issues like abdominal pain, nausea, vomiting, and diarrhea. Moderate illness included pneumonia symptoms with normal blood gases and interstitial ground-glass opacities on high-resolution CT scans. Severe illness was characterized by pneumonia with hypoxemia (peripheral oxygen saturation <92% in ambient air). Critical illness involved ARDS, coagulation disorders, cardiac failure, acute renal injury, and shock. We followed up on the outcome of the patients (death, recovery, number of days spent in the coronary care unit, number of hospitalization days, admission to the hospital intensive care unit), the possible correlations between clinical and paraclinical parameters, and the clinical outcome of the patients and made a comparison between groups of the negative patients and the positive patients.

Ethical consideration

Our study conformed to good clinical practice guidelines and followed the recommendation of Helsinki Declaration. The local ethics review board of the University and Emergency Hospital Bucharest, Bucharest, Romania approved the study protocol (approval number: 37003).

Statistical analysis

We used IBM SPSS Statistics software, version 20.0 (IBM Corp., Armonk, NY). The variables with a continuous Gaussian distribution were expressed as mean ± standard deviation, while the discontinuous variables were expressed as absolute values or as percentages. We used the t-test to compare the means of the values from two subgroups. Pearson's correlation coefficient was used for data with normal distribution. For the prediction analysis, we used logistic regressions (for prediction analysis and modeling binary outcomes). Multiple regression was then utilized to identify subsets of predictive factors, enabling a more detailed understanding of how different variables contribute to the outcomes and helping to isolate the most significant predictors. Statistical significance was defined as a p-value <0.05.

## Results

We identified 33,373 patients hospitalized in our hospital between 1^st^ May 2020 and 30^th^ April 2021. We then reviewed the records of 198 patients who were admitted to our hospital and were diagnosed with VTD, either at admission or during their stay. Of those, 165 were hospitalized in the cardiology department, and 33 were diagnosed with VTD while being hospitalized in other departments of our hospital (internal medicine, gastroenterology, nephrology, neurology, hematology, surgery, orthopedics, obstetrics, and gynecology, etc.). 

**Figure 1:**
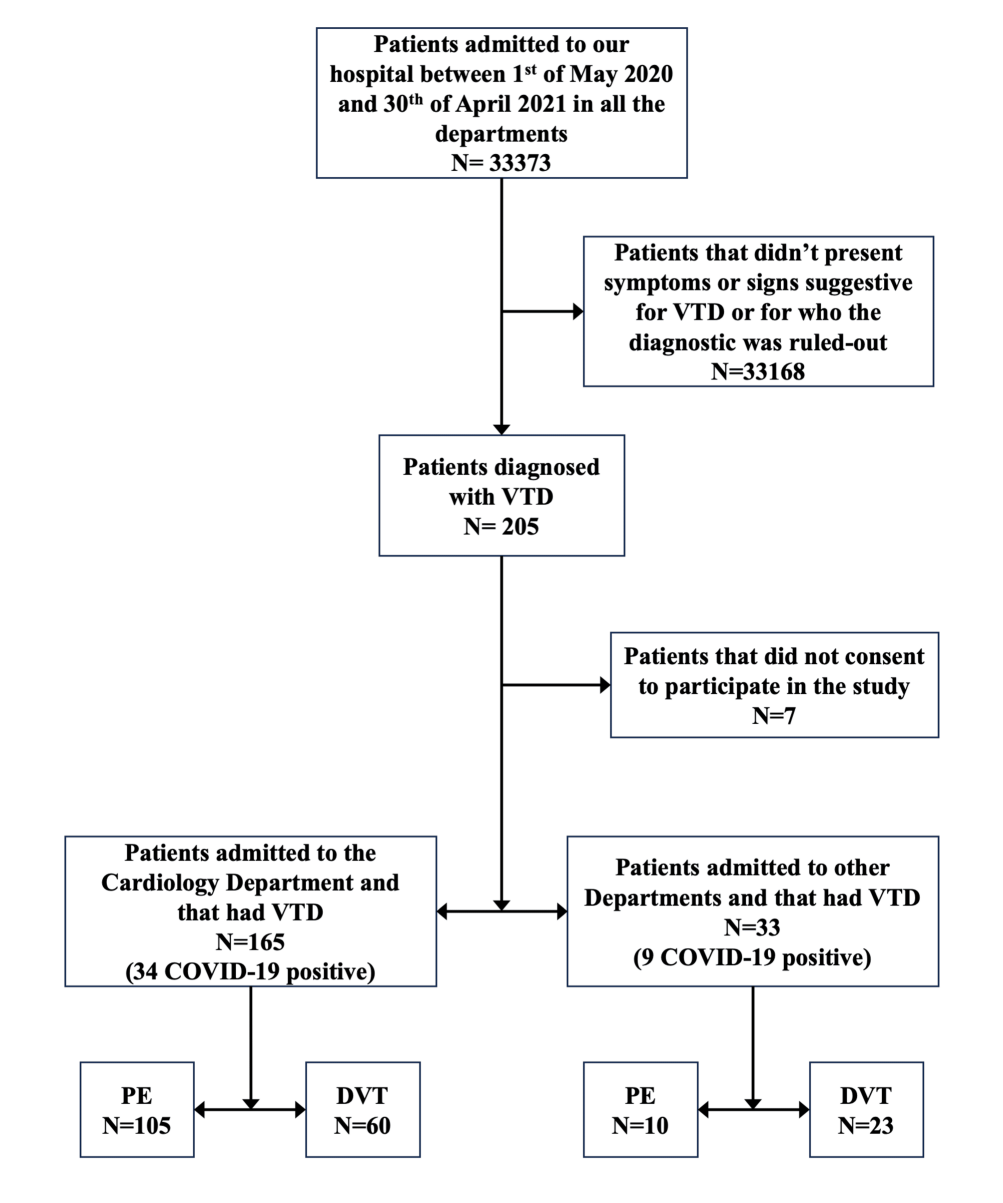
Patient diagnosis flowchart VTD: venous thromboembolic disease; PE: pulmonary embolism; DVT: deep venous thrombosis

Of the patients admitted to the cardiology department, 105 were diagnosed with pulmonary PE (of whom 58 had associated DVT), and 60 patients were diagnosed with isolated DVT. Of the patients admitted to other departments, 10 were diagnosed with PE (eight with concomitant DVT) and 23 were diagnosed with DVT (Figure 1).

Overall, there were 43 patients positive for the SARS-CoV-2 infection (21.7 % of all the VTD patients), of whom 34 (79.9%) were admitted to the cardiology department and nine (21.1%) in other departments. Of the COVID-positive patients, 29 had PE, and 21 had DVT (seven patients having both PE and DVT). Thirty-four patients were positive on the first RT-PCR test and nine on the re-test. The second positive RT-PCR test was not due to the in-hospital spread of the virus. Of the positive patients, 12.2% had mild COVID-19, 61% had a medium form, and 26.8% had a severe form of the disease. 

Clinical, demographic, biological, and echocardiographic characteristics, as well as medical history and thromboembolic risk factors of all patients, are presented in Tables 1-2.

**Table 1:** Comparison between clinical, biological, and imagistic parameters in positive patients and negative patients Group 1: SARS‑CoV‑2-positive test at admission; Group 2: SARS‑CoV‑2-negative at admission. TAPSE: tricuspid annular plane systolic excursion; RV-RA gradient: right ventricle to right atrium gradient; LVEF: left ventricle ejection fraction; HR: heart rate; SBP: systolic blood pressure; DBP: diastolic blood pressure; BMI: body mass index; SaO2: arterial oxygen saturation; CRP: C-reactive protein; PESI: pulmonary embolism severity index; NTproBNP: N-terminal-pro-B-type natriuretic peptide; AST: aspartate aminotransferase; ALT: alanine aminotransferase; BUN: blood urea nitrogen; LDH: lactate dehydrogenase; ARDS: acute respiratory distress syndrome

Parameter		Group 1	Group 2	P-value
Echocardiographic parameters	TAPSE	20.1±5.2	20.3±4.2	0.8
	RV-RA gradient	27.2±20.8	25.5±15.7	0.8
	LVEF mean (%)	53.5±8.4	53.3±11	0.9
	LVEF reduced (<40%)	2.9%	7%	0.7
	LVEF mid-range (³40% <50%)	11.4%	7%	0.7
	LVEF preserved (³50%)	85.7%	86%	0.7
Clinical parameters	HR at admission (beats per minute (bpm))	106.6±30.2	95.9±20	0.009
	HR mean (bpm)	91.1±23.6	81.8±16.5	0.006
	SBP at admission (mmHg)	133.2±20.9	130.5±23.6	0.5
	SBP mean (mmHg)	124.4±13.8	121.4±15.7	0.2
	DBP at admission (mmHg)	79.1±10.8	77.4±13.6	0.4
	DBP mean(mmHg)	74±8.2	77.5± 50.4	0.6
	BMI (body mass index) kg/m2	29.6±7.3	28±5.5	0.3
	Bronchial rales (%)	27.9	9.7	0.002
	Crackles (%)	4.7	5.8	0.77
	Low-grade fever (%)	42.4	11.4	<0.001
	High-grade fever (%)	25.7	7.8	0.004
	Highest temperature during hospitalization (Celsius grades)	37.1±0.9	36.2±3.6	0.2
	Arterial oxygen saturation at admission (SaO2) (%)	93.3±6.1	93.4±10.2	0.1
	Cough (%)	50	7.1	<0.001
	Myalgia (%)	17.1	2.7	0.002
	Dyspnea	68.4	52.2	0.08
PESI score		98.5±34.1	98.5±34.1	0.1
Biological parameters	Admission hemoglobin level (g/dl)	12.6±2.2	13.3±10.2	0.66
	Leukocytes at admission (x10^3^/ul)	12.6±5.6	11.3±7.1	0.02
	Highest leukocyte value (x10^3^/ul)	14±5.3	12.4±6.7	0.19
	Thrombocytes at admission (x10^3^/ul)	244.7±84.4	235.9±99.8	0.6
	Lowest thrombocyte value (x10^3^/ul)	203.57±72.7	205±96.1	0.93
	Highest C-reactive protein (CRP) (mg/dl)	21.4±31.4	13.3±20.8	0.09
	Highest fibrinogen (mg/dl)	517.4±177.1	462.6±182.7	0.09
	Highest troponin I (ng/ml)	1530.2±6782.5	432±864.2	0.45
	Admission NTproBNP (pg/ml)	5363.3±7650.3	4055.2±5794.5	0.43
	Admission aspartate aminotransferase (AST) (mg/dl)	73.6±93.8	47±94.8	0.11
	Admission alanine aminotransferase (ALT)(mg/dl)	63±69.1	47.2±84.1	0.26
	Admission urea (BUN) (mg/dl)	63.5±42.3	52±37.1	0.11
	Admission creatinine (mg/dl)	1.58±2.1	1.2±1	0.09
	Admission creatlne kinase (CK) (U/L)	196.4±293.5	133.6±240.5	0.15
	Admission creatlne kinase myocardial band (CK-MB) (U/L)	22.7±26.7	21±12.2	0.69
	Admission lactate dehydrogenase (LDH)(U/L)	362.1±169.3	304.5±201	0.18
Pulmonary imaging	Accentuation of the interstitial pattern on chest X-ray (%)	34.9	32.9	0.8
	ARDS	11.6	0	<0.001
	Pulmonary condensation or alveolitis on the chest X-ray (%)	2.3	1.9	0.9
	Ground glass image on thoracic CT scan (%)	53.5	16.1	<0.001
	Subpleural condensation on thoracic CT scan (%)	56.7	20.4	<0.001

**Table 2:** Comparison between medical history and thromboembolism risk factors in positive patients and negative patients Group: SARS‑CoV‑2-positive test at admission; Group 2: SARS‑CoV‑2-negative at admission; COPD: chronic obstructive pulmonary disease

Parameters	Group 1	Group 2	p-value
Former smoker	34.1%	32.6 %	0.85
Current smoker	22%	22.7%	0.9
Stroke history	24.4%	11.1%	0.03
Congestive heart failure	10%	14.1%	0.5
Hypertension	61%	65.5%	0.5
Dyslipidemia	65%	60.6%	0.6
Coronary disease	7.3%	10.2%	0.58
Valvular disease	9.8%	16.5%	0.28
COPD	7%	4.5%	0.51
Peripheral arterial disease	0%	2.6%	0.29
Chronic kidney disease	14%	10.5%	0.52
Neoplasm	9.3%	22.6%	0.053
Recent surgery	9.3%	14.2%	0.4
Thrombophilia	4.7%	1.3%	0.16
Chronic venous insufficiency	9.3%	9.7%	0.94
Recent fracture	2.3%	6.5%	0.29
Bed immobilization	9.3%	12.9%	0.52
Dementia	11.6%	9.7%	0.7
Pregnancy	0%	2.6%	0.29
Childbed	0%	2.6%	0.29
History of thromboembolism		13.5%	0.65

Comparison between SARS-CoV-2-positive patients and SARS-CoV-2-negative patients

Demographic Parameters

In the whole study group, the mean age was 63.2±15.3 years (20 to 93 years), with 51.5% being females and 48.5% being males. In the COVID-negative subgroup, the mean age was 62.7 ±15.8 years, with 54.8% females and 45.2% males, whereas in the COVID-positive subgroup (mean age 65.2±13.6, 31 to 88 years), a higher percentage was male (60.5%).

Clinical Parameters

The mean interval between COVID-19 diagnosis and hospitalization for VTD was 7.0±22.1 days (maximum 180 days). The COVID-positive patients had a significantly higher heart rate (106.6±30.2 beats per minute (bpm)) and mean heart rate (91.1±23.6 bpm) during hospitalization than the negative patients (95.9±20.0 and 81.8±16.5 bpm) (p = 0.009 and p = 0.006, respectively). 

The COVID-positive patients had, as expected, statistically significant higher rates of cough (50% vs. 7.1%, p<0.001), myalgia (17.1% vs. 2.1%, p = 0.002), bronchial rales (27.9% vs. 9.7%, p = 0.002), low-grade fever (42.4% vs. 11.4%, p<0.001), and high-grade fever (25.7% vs. 7.8%, p = 0.004).

Interestingly, we found no significant differences in the PESI score between COVID-positive (110.7±28.9) and COVID-negative patients (98.5±34.1, p = 0.09). Also, the COVID-positive patients didn’t experience dyspnea in a more significant way (68.4% vs. 52.2%, p = 0.08) (Table 1). Different clinical parameters were analyzed according to the available definitions [7-10].

Comparison of medical history and thromboembolic risk factors between COVID-positive and COVID-negative patients

We analyzed the most frequent predisposing factors for VTD, as well as the medical history of the patients. The most frequent comorbidities in both groups were hypertension and dyslipidemia, followed by diabetes mellitus and chronic kidney disease. Twenty-four (55.8%) of the COVID-positive patients and 69 (44.5%) of the COVID-negative patients had no known predisposing factors for venous thromboembolism.

Comparison of biological parameters between COVID-positive and COVID-negative patients

The COVID-positive patients showed a higher number of leukocytes at admission (p = 0.02), as well as more pronounced neutrophilia (p = 0.001) and lymphopenia (p = 0.002). 

We found no other significant differences regarding inflammatory markers or kidney and liver function (Table 1). Although COVID-positive patients had higher values of NTproBNP than negative patients, the difference did not reach statistical significance (p = 0.43). 

Interestingly, we found that the COVID-positive patients had lower values of high-sensitivity cardiac troponin (hs-cTnT) than the negative ones, but without statistical significance. When considering blood gas parameters, the two subgroups showed similar values. 

Anticoagulant therapy, hemorrhagic events, and other medical treatments

On the day of hospital admission, a higher percentage of COVID-positive patients were already taking oral anticoagulants compared to the COVID-negative ones (19.4 vs. 10.9%, p = 0.18). The COVID-negative patients were receiving mostly anti-vitamin K (VKAs), or apixaban, whereas the positive patients were taking apixaban or low molecular weight heparin (LMWH). 

Regarding the indication of thrombolysis for high-risk PE, 30 (19.4%) of the COVID-negative patients and eight (18.6%) of the COVID-positive patients received fibrinolytic therapy with alteplase (p = 0.86). 

At hospital discharge, from the COVID-negative-patient subgroup, 38.9% received apixaban, 40.5% received rivaroxaban, 6.9% received LMWH, and 6.1% received VKAs, whereas in the COVID-positive-patient subgroup, 45.7% received apixaban, 40% received rivaroxaban, 8.6% received LMWH, and only 2.9% received VKAs.

There were no statistically significant differences between the two subgroups regarding the use of aspirin (16.1% in the negative subgroup vs. 20.9%, p = 0.54), clopidogrel (4.5% vs. 2.3%, p = 0.49), or dual antiplatelet therapy (1.3 vs. 2.3, p = 0.65). 

During hospitalization, hemorrhagic events occurred in a similar proportion in the two study groups, most of them being minor. Of the COVID-positive patients, five (11.6%) experienced bleedings, two of them being minor, two of them being upper gastrointestinal bleedings that were treated conservatively, and one hemoptysis requiring vascular embolization. The COVID-negative patients required positive inotropic medication more than COVID-positive patients (15.9% vs. 7.3%, p = 0.16). 

Antibiotic therapy was administered to 60.5% of the COVID-positive patients and 38.9% of the COVID-negative patients. As specific COVID-19 medications recommended by the guidelines in practice at the moment of admission, COVID-positive patients received: azithromycin (20.9%), corticosteroids (15.1%), and interleukin inhibitors (35.7%). 

Pulmonary imaging and lower-extremity duplex venous ultrasound

As expected, there were significant differences between the two subgroups regarding pulmonary imaging. Among COVID-positive patients, 11.6% had a chest X-ray suggestive of ARDS compared to none of the COVID-negative patients (p<0.001). 

Furthermore, the pulmonary CT scan of the COVID-positive patients showed ground-glass images (53.5%) and subpleural condensation (56.7%) in a significantly larger proportion than that of the COVID-negative patients (16.1% and 20.4%; p<0.001 for both). The mean lung parenchymal involvement in the COVID-positive patients was 50.4±30.1%. 

**Figure 2:**
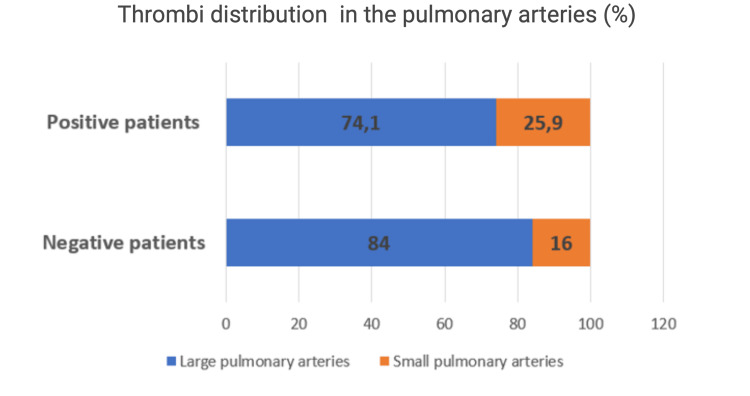
Thrombi distribution in the pulmonary arteries in the two groups

Although there were no significant differences regarding the distribution of the thrombi in the pulmonary arteries (large arteries defined as the pulmonary trunk, left and right pulmonary arteries, and lobar pulmonary arteries, and small arteries defined as segmental and subsegmental pulmonary arteries), there was a tendency towards affecting the small arteries in the COVID-positive-patients subgroup (25.9% vs. 16% in the COVID-negative-patients subgroup) (Figure 2). Also, a higher percentage of COVID-positive patients (13.9%) than COVID-negative ones (9.0%) (p = 0.47), had a thrombus at the bifurcation of the pulmonary artery bifurcation (saddle-like thrombus). 

Lower-extremity venous Doppler was performed in all COVID-positive patients who were diagnosed with DVT, and all of them presented with thrombi in the iliofemoral veins. Of the COVID-negative patients, the majority had proximal thrombosis, and only four had distal venous thrombi. 

Echocardiographic parameters 

There were no significant differences concerning the mean LVEF between the COVID-positive patients and COVID-negative patients. Also, we found no significant differences between the right atrioventricular pressure gradient of COVID-positive and COVID-negative patients (27.2±20.8 vs 25.5±15.7 mmHg, p=0.74) or between TAPSE (20.0±5.2 vs 20.3±4.2, p=0.79). Also, 11.6% of the COVID-positive patients and 11% of the COVID-negative patients had a paradoxical movement of the interventricular septum (p = 0.9). 

Intensive care unit hospitalization, duration of hospitalization, and evolution and prognosis

A higher percentage of COVID-positive patients compared to COVID-negative patients required admission to the intensive care unit, but no statistical significance was reached (16.7% vs. 9.8%, p = 0.36). The mean stay in the intensive care unit was also longer for COVID-positive patients than for COVID-negative patients (3.0±4.6 vs. 1.4±2.2 days, p = 0.01). Overall, COVID-positive and COVID-negative patients had a similar duration of hospitalization (9.8±6.8 vs. 9.3±9.3 days, p = 0.76). A higher proportion of COVID-positive than COVID-negative patients with DVT died during hospitalization, but the number was low in both groups and therefore not reach statistical significance. 

Correlations 

We tried to identify correlations between major adverse cardiovascular events (mainly death during hospitalization) and clinical and paraclinical parameters. We analyzed the correlations separately for the COVID-positive and COVID-negative patients. 

SARS-CoV-2-Positive Patients

**Figure 3:**
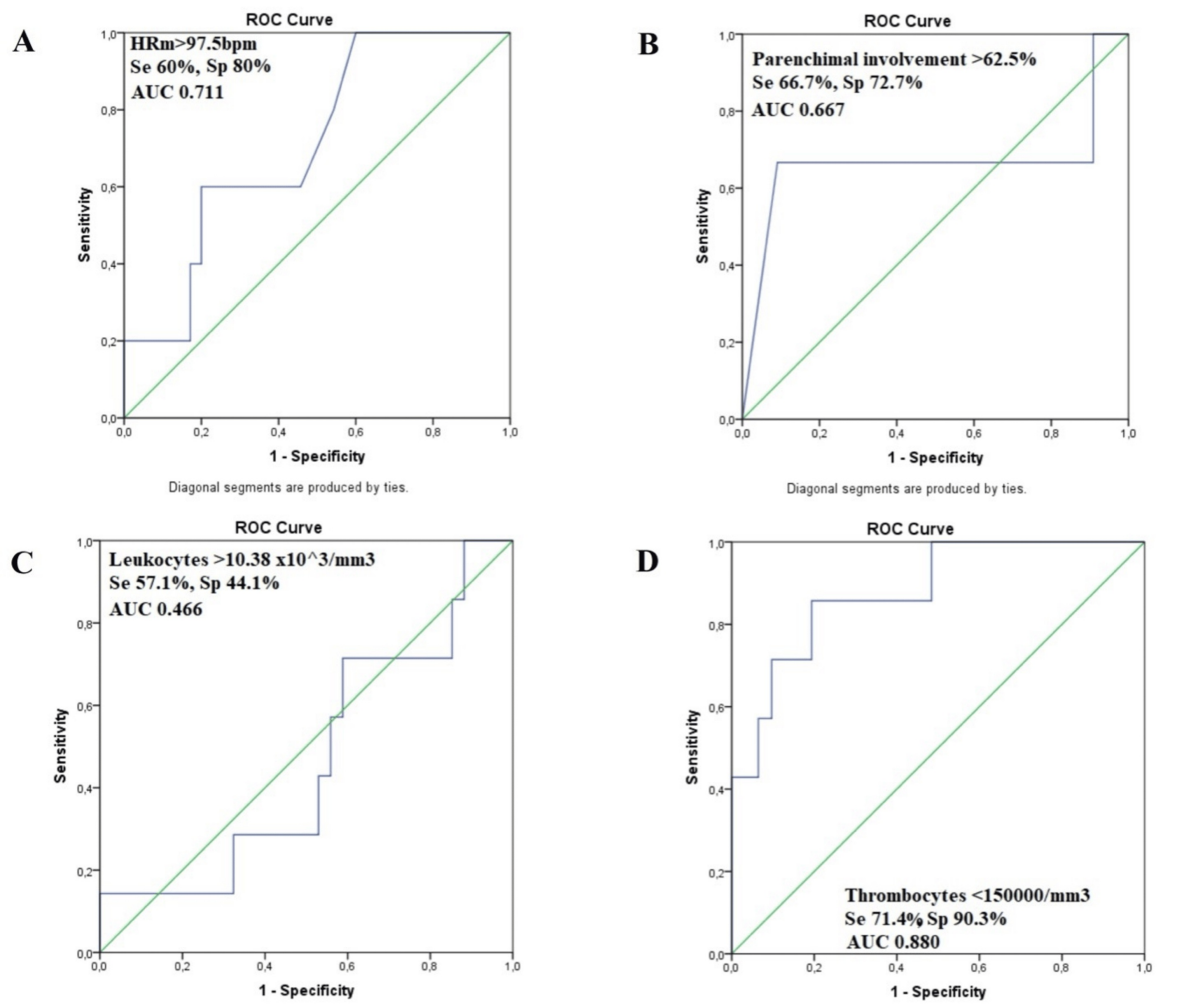
The ROC curves for different parameters and in-hospital death for the positive-patients subgroup Panel A: ROC curve for heart rate mean (HRm) during hospitalization and the in-hospital death of the patients (AUC = 0.711, CI 0.49-0.92). Panel B: ROC curve for the parenchymal involvement percent on the thoracic computed tomography hospitalization and the in-hospital death of the patients (AUC = 0.667, CI 0.19-1). Panel C: ROC curve for the highest leukocyte value and the in-hospital death of the patients (AUC=0.466, CI 0.23-0.7). Panel D: ROC curve for the minimum thrombocyte number and the in-hospital death of the patients (AUC=0.563, CI 0.412-0.713). ROC: receiver operating characteristic; AUC: area under the curve; Se: sensitivity; Sp: specificity

For COVID-positive patients, the vast majority of correlations were weak, probably because of the very low number of deaths (n = 4), but death was associated with the length of stay in the intensive care unit (p<0.001, R = 0.592), with hypotension at admission (p<0.001, R = 0.370), with the mean HR during hospitalization (p = 0.003, R = 0.343), with the maximum number of leukocytes during hospitalization (p = 0.011, R = 0.409), with the LDH value (p = 0.047, R = 0.393), and inversely correlated with the minimum number of thrombocytes during hospitalization (p = 0.001, R = -0.517), and the partial pressure of the oxygen at admission (p = 0.012, R = -0.478) (Figure 3). 

In addition, when plotting the receiver operating characteristic (ROC) curves, the lower the minimum value of thrombocytes during hospitalization, the bigger the risk of death was (area under the curve (AUC) = 0.880, CI 0.743-1 when thrombocytes<150000/mm^3^ with sensitivity 71.4% and specificity 90.3%). The minimum number of thrombocytes during hospitalization was also correlated with the number of days spent in the hospital by COVID-positive patients. No predictive model was obtained because the low number of deaths didn’t allow a qualitative statistical analysis.

SARS-CoV-2-Negative Patients 

**Figure 4:**
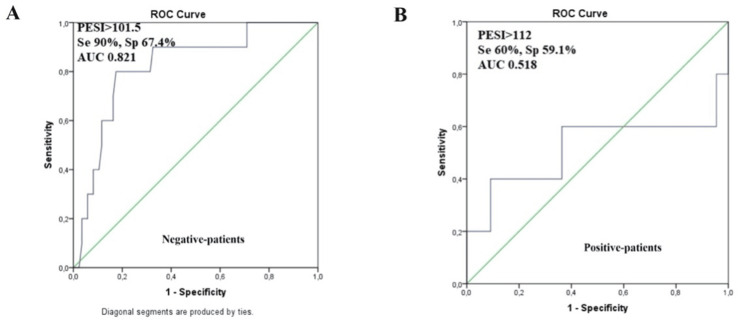
The ROC curves for the PESI score and in-hospital death for the negative and the positive patients Panel A: ROC curve for the PESI score and the in-hospital death of the negative patients (AUC = 0.821, CI 0.689-0.952). Panel B: ROC curve for the PESI score and the in-hospital death of the positive patients (AUC = 0.518, CI 0.141-0.895). ROC: receiver operating characteristic; PESI: pulmonary embolism severity index; AUC: area under the curve; Se: sensitivity; Sp: specificity

For COVID-negative patients, death was moderately correlated with the value of the systolic blood pressure at admission (p<0.001, R=-0.384), mean of the systolic blood pressures during hospitalization (p<0.001, R=-0.432), hypotension at admission (p<0.001, R=0.408), PESI score (p<0.001, R=0.358), maximum leukocyte value (p<0.001, R=0.393), minimum thrombocyte number (p<0.001, R=-0.316), lactate dehydrogenase (p<0.001, R=0.373). No predictive model was obtained because the low number of deaths didn’t allow a qualitative statistical analysis. The ROC curve for the PESI score (AUC=0.821, CI 0.689-0.952) (Figure 4) showed a good predictive power for in-hospital death. 

## Discussion

Currently, it is widely accepted that SARS-CoV-2 infection is associated with an increased risk of thromboembolic disease, both arterial and venous, and with a specific kind of vasculopathy. Of note, we relied on established definitions from the literature to define and report our results, particularly regarding the PESI score, fever, smoking status, and the severity of the SARS-CoV-2 infection [8-11]. 

In the vasculopathy associated with COVID-19 disease, microvascular damage was observed, with thickening of the vascular wall and microthrombi formation [12]. This is caused by a combination of factors, such as the severe inflammatory response of the organism to the infection, disseminated intravascular coagulation, dysregulation of the complement system, as well as a possible virus-induced local inflammatory reaction and cytopathic effect that may affect endothelial cell function. Hypoxemia (which also induces heparinase activity) is another probable mechanism that increases the risk of thrombosis in COVID-19 patients [6].

Although still in debate, the current guidelines recommend prophylactic or therapeutic doses for anticoagulation with LMWH in all patients hospitalized for COVID-19. The American Society of Hematology recommends the use of therapeutic-dose heparin for patients who have a D-dimer above the upper limit of normal, require low-flow oxygen, and have no increased bleeding risk [13]. Hospitalized, nonpregnant adults who are receiving intensive care unit level of care (including patients who are receiving high-flow oxygen) should receive prophylactic-dose heparin as venous thromboembolism prophylaxis unless a contraindication exists [13]. 

Still, some studies have reported that more than half of the thromboembolic events were diagnosed in the first 24 hours after hospital admission and therefore might not be preventable only by in-hospital thromboprophylaxis [14]. Studies showed that oral anticoagulation therapy did not influence the risk of ARDS or death in patients hospitalized with COVID-19. In our study, 19% of patients with VTD were on oral anticoagulants before admission, 13% with non-VKAs, and 8% with VKAs [15]. 

We observed a higher bleeding rate in COVID-positive patients, with one patient presenting severe hemoptysis and requiring endovascular embolization. This is important, considering the fact that all patients received the same anticoagulation regimen and that the two subgroups didn’t present significant differences regarding the use of medication that facilitates bleeding, such as antiplatelet or anticoagulation medication. Moreover, this may point out that the SARS-CoV-2 infection may be considered a bleeding predisposing factor under certain circumstances.

The reason why both prophylactic as well as therapeutic doses of anticoagulants are ineffective in preventing VTD in some cases could be that the coagulopathy present in the COVID-19 disease is caused by a connection between clotting system activation and immuno-mediated inflammatory response. Consequently, acting only upon one pathway is not enough to stop the process [15]. 

In our study, the mean age in the COVID-positive patients presenting with VTD was 65.2±13.6, which mirrors the data present in the literature, but with a higher percentage of male patients (60.5%) [16]. The mean period between COVID-19 infection and hospital admission for VTD was 7.0±22.1 days, which is similar to other reports [16]. 

Although the differences did not reach statistical significance, a higher mortality was observed in the COVID-positive-patients subgroup. Still, this may be due to the overall low mortality. This appears to be caused by the pulmonary parenchymal involvement and other systemic complications rather than reflecting a bigger severity of VTD, as we found no significant differences between the two subgroups regarding the PESI score (although COVID-positive patients had slightly higher scores), hypotension at admission, or the necessity to administer positive inotropic medication. Also, in our study, on blood gas analysis, COVID-positive patients had similar oxyhemoglobin saturations as the COVID-negative patients.

As expected, COVID-positive patients had more COVID-19-specific symptoms. In terms of pulmonary imaging, we observed a tendency for a more peripheral distribution of the pulmonary thrombi in COVID-positive patients, as was also reported by other studies [12]. 

Interestingly, most patients in both COVID-positive and COVID-negative subgroups had no known predisposing factors for venous thromboembolism. This could recommend the severity of the SARS-CoV-2 infection as an important predisposing and causative factor for VTD but also might reflect the pandemic reality in which hospital stays were shortened to the maximum, leaving potential causes such as obscure neoplasia undiscovered.

The minimum value of thrombocytes during hospitalization was inversely correlated with the risk of death in our studied COVID-positive patients. The minimum number of thrombocytes during hospitalization was also correlated with the number of hospitalization days in the positive group. Therefore, a low number of thrombocytes is associated with a worse outcome in COVID-19 patients hospitalized with VTD. This is in accordance with other data in the literature. For example, Guan et al. found that thrombocytopenia was identified in up to 57.7% of severe cases of COVID-19 patients in comparison with 31.6% of patients with the milder form of the disease [17]. 

As expected, for COVID-negative patients, the PESI score proved to have a high predictive value for the clinical outcome of COVID-negative patients, far better than in the case of COVID-positive patients, whereas severity of the disease is an important factor not included in any risk score. Of note, the PESI score had not yet been validated in the PE associated with the COVID-19 infection, and some studies point out that it may not be as accurate as in COVID-negative patients. The vast majority of the COVID-19-positive patients admitted to our hospital with PE or DVT had at least a moderate severity of the disease. Also, the mean pulmonary involvement on the CT scan was around 50%. These two findings support the idea that in the case of COVID-19 and PE, the PESI score should be completed by an accurate evaluation of the severity of the disease to predict outcomes and that the severity of COVID-19 is a predictive factor for VTD occurrence.

Currently, although the incidence of DVT might be higher than that reported, the guidelines of the American College of Chest Physicians and the guidelines of the International Society on Thrombosis and Hemostasis do not recommend a routine screening ultrasound for the detection of DVT in COVID-19 patients [18]. Therefore, we did not perform routine lower-extremity ultrasonography on our patients unless they showed signs and symptoms of DVT. Of importance, many published studies plead for the “in-situ” formation of the thrombi in the pulmonary arteries, which might explain the lack of deep venous thrombosis in some patients with pulmonary embolism and COVID-19 infection. No considerations can be made regarding the location of the thrombi in the patients with DVT, as there is a tendency to hospitalize only the patients with proximal thrombi while ambulatory treating the patients with distal DVT.

Many studies suggest that D-dimers should not be used for the diagnosis of VTD in hospitalized COVID-19 patients because the value of this parameter is increased significantly due to the infectious disease. On the other hand, some authors advocate for the usefulness of the D-dimer value in predicting mortality in COVID-19 [19, 20]. The present study did not evaluate this hypothesis, as only semi-quantitative D-dimer kits were available on-site. 

Although higher NTproBNP values were observed in the COVID-positive-patient subgroup, statistical significance was not reached, similar to the information provided by other studies. 

We found no significant differences concerning the systolic function of the left ventricle between COVID-positive patients and COVID-negative patients, which is in accordance with other data published in the literature [21]. This might imply a similar impact of the pulmonary thrombi on the heart function in the COVID-19-positive and negative patients. Moreover, we found no significant differences between the right atrioventricular pressure gradient of COVID-positive and COVID-negative patients or the longitudinal function of the right ventricle, which differs from previously reported data [21]. One explanation for this may lie in the selection of the cohorts. 

Study limitations and future perspectives

The major limitation of this study is its retrospective nature and the consequently in-homogenous evaluation of patients. In addition, although it presents the experience of the biggest emergency hospital in Romania, the risk of selection bias persists, as the patient's addressability was reduced due to the restrictions and for fear of the in-hospital spread of the disease. Another limitation of the study lies in the small number of deaths, which interfered with the possibility to perform important prediction analysis. We did not include the patients who had recently recovered from SARS-CoV-2 infection (less than one month), due to the small number of patients in this subgroup (n = 5) and the low probability of reaching statistical significance. Still, we acknowledge the importance of this subgroup of patients as a cornerstone of the investigation of the link between SARS-CoV-2 infection and thromboembolic disease. Our data should be completed by data from the other pandemic waves or post-pandemic seasonal outbursts of COVID-19, which we aim for as a future perspective.

## Conclusions

The present article summarizes the main characteristics of VTD observed in patients hospitalized in a multidisciplinary emergency hospital during the first two waves of the COVID-19 pandemic while comparing clinical and paraclinical parameters of positive and negative patients for the SARS-CoV-2 infection. Patients with severe forms of the disease remain at high risk of developing VTD and have a high risk of negative outcomes despite prophylactic anticoagulation. In COVID-19-infected patients, the PESI score is not as good a predictive tool as in the COVID-negative patients. Although specific risk scores are still to be developed for COVID-19 patients with VTD, the occurrence of low thrombocyte count during hospitalization is associated with poor prognosis. Special attention should be given to any change in the clinical picture of the patients, with prompt diagnosis and treatment measures taken when hemodynamic deterioration or accentuation of dyspnea is observed. While the acute phase of the pandemic may have ended, the lessons learned about VTD associated with SARS-CoV-2 infection remain relevant for long-term public health, patient care, and future preparedness efforts.
